# Squamous cell carcinoma of the pancreas

**DOI:** 10.3747/co.v15i6.265

**Published:** 2008-12

**Authors:** A. Al-Shehri, S. Silverman, K.M. King

**Affiliations:** * Department of Medical Oncology, Cross Cancer Institute, Edmonton, AB; † Department of Lab Medicine, Misericordia Hospital, Edmonton, AB

**Keywords:** Squamous cell carcinoma, pancreatic malignancies, chemotherapy

## Abstract

Pancreatic malignancies can be subdivided into endocrine and non-endocrine processes. Of the non-endocrine tumours, ductal carcinoma is the most common, and the ductal carcinomas can be further subdivided into adenocarcinomas and squamous cell carcinomas. The adenocarcinomas constitute most of the non-endocrine pancreatic malignancies, and the treatment options for these, although limited in efficacy, are relatively well established. The squamous cell carcinoma pathology is a rare entity, and few reports of it are found in the literature. As a result, treatment options for squamous cell carcinoma of the pancreas are poorly understood. Here, we report the presentation of a 48-year-old woman with metastatic squamous cell carcinoma of the pancreas. The subsequent investigations, treatment, and outcome are described.

## 1. INTRODUCTION

Pancreatic squamous cell carcinoma is an extremely rare subtype of pancreatic cancer of ductal origin. It is poorly understood with respect to causation, risk factors, biologic behaviour, and response to chemotherapeutic agents. Here, we present the case of a 48-year-old woman with a diagnosis of pancreatic squamous cell carcinoma, with a review of the existing literature, including case reports and review articles.

## 2. CASE PRESENTATION

A 48-year-old woman presented to her local emergency department on July 9, 2006, complaining of fatigue, anorexia and weight loss, nausea and vomiting, and upper abdominal and back pain. These symptoms had all developed in rapid succession over the course of 3 weeks. She had also noted jaundice. Investigations done at the time included a normal complete blood count and elevated bilirubin of 100 μmol/L. The woman was treated for the pain and nausea, and an outpatient abdominal ultrasound was booked for July 24, 2006.

Ultrasound showed dilatation of both the common bile duct (17 mm) and the pancreatic duct (5 mm). The head of pancreas could not be evaluated. A slight contour deformity measuring 3.5 × 3.5 × 2.0 cm was also seen in the left hepatic lobe. Computed tomography (ct) imaging was recommended. On August 3, 2006, the patient underwent triphasic abdominal ct for further evaluation of her hepatopancreaticobiliary anatomy. Enlargement of the pancreatic head and uncinate process (measuring 4.4 × 4.2 cm) was observed at this time. No calcification or hyper-enhancement was identified. The enlarged pancreatic head was obstructing both the pancreatic and the common bile duct and was abutting the superior mesenteric vein on the right to almost 180 degrees. The ct results also showed dilatation of both the biliary and the pancreatic duct, two hepatic lesions (both in segment 3), and a 14-mm hypodense nodule in the spleen.

A provisional diagnosis of pancreatic carcinoma with liver metastasis was postulated. The patient underwent endoscopic retrograde cholangiopancreatography on August 14, 2006, for palliative decompression of the biliary and pancreatic ducts. This procedure failed secondary to significant narrowing of the second portion of duodenum.

**FIGURE 1:**
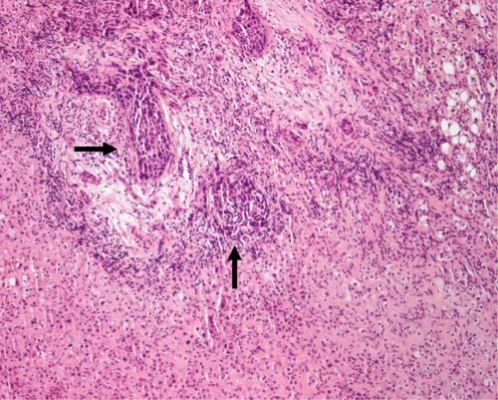
Hepatic biopsy showing normal liver in lower quadrant, with neoplastic clusters (arrows) of metastatic squamous cell carcinoma (hematoxylin and eosin stain, 20x× magnification).

The patient was admitted (same day) to hospital for consideration of palliative gastrojejunostomy and choledochojejunostomy. Examination on admission showed a jaundiced lady with normal vital signs. Respiratory and cardiac examination was unremarkable. Abdominal examination showed a palpable mass in the right upper quadrant along the liver edge. Laboratory investigations revealed a bilirubin of 321 μmol/L, alanine transaminase of 247 U/L, alkaline phosphatase of 900 U/L, and a prothrombin time (international normalized ratio) of 1.6. The patient went to the operating room on August 16, 2006. She underwent gastrojejunostomy, choledochojejunostomy, open cholecystectomy, and hepatic lesion biopsy. The primary tumour was left in place. Pathology from the hepatic biopsy showed metastatic poorly differentiated squamous cell carcinoma (Figure 1). Desmoplastic stroma surrounding neoplastic clusters was noted. The patient was discharged from hospital within 1 week and was referred to both medical oncology and palliative care.

On September 19, 2006, the patient was seen in a medical oncology clinic, where she underwent further evaluation to rule out other primary sources of squamous cell carcinoma. A 35 pack–year history of smoking was noted. Physical examination also revealed a small left mandibular mass (2 × 2 cm) of 6 months’ duration, and a slight facial deviation. Given the history and physical exam, concern arose with respect to a lung or head-and-neck primary. However, imaging of head, neck, and chest by ct on October 4 revealed no intracranial pathology, and no suspicious pulmonary nodules were noted. The patient underwent core biopsy of the mandibular lesion, which showed a benign intravascular pyogenic granuloma.

The final diagnosis was squamous cell carcinoma of the pancreas with metastatic disease to liver. The decision of the gastrointestinal tumour group was to treat with palliative chemotherapy using a lung chemotherapy protocol of gemcitabine and carboplatin. The patient underwent 2 cycles of chemotherapy, but died November 15, 2006.

## 3. DISCUSSION

The pancreas can be involved by a variety of neoplastic processes, both endocrine and non-endocrine. Endocrine tumours can originate from the islets of Langerhans. Non-endocrine tumours can be classified into the following histologic subdivisions 1–3:

- Duct cell origin
- Acinar cell origin
- Mixed cell type
- Connective tissue origin
- Uncertain histogenesis

Ductal carcinoma is the most common pancreatic cancer, and this classification is further subdivided into adenocarcinoma and (the rare) squamous cell carcinoma. The classic adenocarcinoma constitutes about 75%–81% of all pancreatic cancers, and so it is by far the most common pancreatic malignancy 1,2,4,5.

Pure squamous cell carcinoma of the pancreas is so rare that is not mentioned in most textbooks dealing with pancreatic malignancies 1. It has various reported incidence rates, ranging from 0.5% to as high as 5% of pancreatic ductal carcinoma 2,4,5–13. Halpert and his group (as quoted by Brown *et al.*) in their series of 120 patients, described an incidence rate of 5% pure squamous pathology 5. However, in a much larger series review such as Baylor and Berg (5075 patients), the reported incidence of pure squamous cell carcinoma was 0.5% of the pancreatic ductal carcinomas 14. Of the 1300 cases of pancreatic cancers observed at autopsy in a survey in Japan in 1992, 0.7% were squamous cell carcinoma 7. However, no cases of squamous cell carcinoma were found in the 1211 pancreatic carcinomas compiled from registries for pancreatic cancer in Japan (as quoted by Anagnostopoulos *et al.*) 8. This discrepancy in the reported incidence rates can be explained in part by the fact that some of the cases represent adenosquamous carcinoma rather than pure squamous cell carcinoma of pancreas 8. Because of its rarity, squamous cell carcinoma of the pancreas is typically diagnosed only after other primary sources have been excluded by appropriate diagnostic tests, and only after the presence of a glandular component has been ruled out using multiple-cut specimens and several histologic techniques 15.

Normally, the pancreas is totally devoid of squamous cells. It is not uncommon to find squamous metaplasia of the ductal columnar cells during periods of inflammation, such as in pancreatitis, and in fact, squamous cell carcinoma of the pancreas has been thought to arise from ductules that have undergone squamous metaplasia secondary to chronic inflammation 3. Atypical squamous epithelium can also be seen in cytologic specimens obtained from pancreatic lesions arising from other than inflammatory conditions—for example, chronic pancreatitis. These conditions may include squamous metaplasia related to pancreatic or biliary duct stent placement, and primary or metastatic squamous cell carcinoma 10. Differentiation between the atypical squamous epithelium of benign conditions and the cytologic aspirates of carcinoma is possible by noting the small number of atypical cells and the lower degree of nuclear atypia found in the former condition 10. Squamous metaplasia of the pancreatic ducts is found in 9%–64% of pancreases routinely examined at necropsy, and despite the relative frequency of squamous metaplasia, transformation to squamous cell carcinoma of pancreas is an extremely rare occurrence^4^–^6^,^8^.

Multiple theories have been postulated to explain the development of pancreatic squamous cell carcinoma. These include 4,7–9,13

- malignant change in a primitive cell capable of differentiating into either squamous or glandular carcinoma,
- squamous change in a pre-existing adenocarcinoma,
- malignant transformation in a squamous metaplasia of the ductal epithelium,
- malignant change in an aberrant squamous cell, and
- the theory of tumour collision.

Clinicopathologic and immunohistochemical studies have helped little in clearing the confusion raised by these theories. Given the absence of any glandular component in the pancreatic specimens of our patient, the first two of the above-mentioned theories are unlikely origins for her pancreatic squamous cell carcinoma.

In a consideration of the various theories of the development of primary pancreatic squamous cell carcinoma, metastatic squamous cell carcinoma of the pancreas should be noted to be a far more common cause of malignant pancreatic squamous cell carcinoma lesions than the development of a primary squamous cell carcinoma. Cubilla and Fitzgerald (as quoted by Layfield *et al.*) in their autopsy study, did not find a primary squamous cell carcinoma of pancreas, but 261 of 411 neoplasms within the pancreas were metastatic, with 49 metastases being from the lung, 12 from the cervix, and 10 from the esophagus 10. Statistically, the presence of pure squamous cell carcinoma in the pancreas favours a metastatic lesion until proven otherwise, and appropriate radiographic and endoscopic evaluations are needed to rule out this possibility.

No specific known risk factors exist for the development of primary squamous cell carcinoma of the pancreas other than the classical risk factors associated with the more common ductal adenocarcinoma. In one of the theories noted earlier, pancreatic squamous cell carcinoma has been postulated to arise from ductules that have undergone squamous metaplasia secondary to chronic inflammation 3, and most, if not all, instances of pancreatic squamous cell carcinoma are associated with chronic pancreatitis 1. Mikal and Campbell, as quoted by Sears *et al.,* reported pancreatitis in 49 of 100 autopsied cases with pancreatic carcinoma, and Gambill, as quoted by Sears *et al.,* showed significant pancreatitis by histologic criterion in 26 of 225 patients with pancreatic and ampullary carcinoma 1. These pathology studies suggest that chronic pancreatitis may be associated with pancreatic carcinoma, but they could not specifically evaluate whether the malignant changes developed from the inflammatory lesions 1.

Calcifications occur in 1%–4% of all pancreatic cancers. Calcifications probably represent foci of hemorrhages that occurred in the core of the mass or episodes of perineoplastic pancreatitis 12. A number of investigators have found that the combination of pancreatic calcification and chronic pancreatitis is associated with pancreatic cancer. Currently, no evidence supports a cause–effect relationship between pancreatitis and carcinoma 1.

Questions have been raised about the relationship between cholelithiasis and pancreatic cancers, and there may be a higher incidence of cholelithiasis in women with pancreatic cancer, but no good existing controlled studies prove this hypothesis 1. Bell, in his review of 609 autopsied pancreatic cancer patients, concluded that carcinoma of the pancreas is unrelated to cholelithiasis 16.

The biologic behaviour of pancreatic squamous cell carcinoma appears to be similar to that of the much more common ductal adenocarcinoma. Both tend to occur in older people, are usually metastatic at the time of diagnosis, respond poorly to chemotherapy and radiotherapy, and are generally associated with very short survival 2,3,13. In an analysis of 25 patients, mean age at diagnosis of pancreatic squamous cell carcinoma was 62 years (range: 33–80 years) 5. Another analysis of 6 patients reported a mean age of 65 years 4. Other studies revealed that 92% of pancreatic squamous cell carcinomas occur in patients over the age of 50 years 2,9,13. The analysis of the 25 cases did not show a significant sex preference (there were 14 men and 11 women in the group) 5, and so pancreatic squamous cell carcinoma seems to affect both sexes equally.

The clinical presentation of pancreatic squamous cell carcinoma is indistinguishable from that of adenocarcinoma, with the most common presenting symptoms being abdominal and back pain, anorexia and weight loss, nausea and vomiting, and obstructive jaundice. Obtaining the exact diagnosis before surgery or autopsy is therefore difficult 2–6,8,9,13. One of the unusual presentations is upper gastrointestinal bleeding and melena secondary to gastric invasion 17.

The anatomic head, tail, and body of the pancreas seem to be affected equally by squamous cell carcinoma 4–6. In one report, tumour was located in the head of the pancreas in 73% of cases, the body in 45%, and the tail in 23% (there is some overlap because the tumour may span more than one portion of the organ) 2. Mean tumour size as reported by Brown *et al.* in the review of 25 cases with detailed data was 7.8 cm 5. Approximately 95% of patients show evidence of disseminated or locally metastatic disease at the time of initial evaluation or laparotomy 2, with the regional lymph nodes, liver, lung, and bones being most commonly targeted 4,13.

No specific laboratory investigation is helpful in the diagnosis or monitoring of pancreatic squamous cell carcinoma. However, two reports discussed the role of squamous cell carcinoma antigen (Ag) in diagnosing and monitoring the disease. Hachiya, as quoted by Minami *et al.,* reported that the serum squamous cell carcinoma Ag level was very high in a patient with pancreatic squamous cell carcinoma 17. Minami *et al.* also reported an elevated level of squamous cell carcinoma Ag (14.9 U/mL) upon diagnosis, which immediately delined to within normal limits after complete resection of the tumour 17. This finding suggests that the level of serum squamous cell carcinoma Ag may be a useful marker for tumour recurrence, but the association requires further validation.

Hypercalcemia is another laboratory finding that has been reported in pancreatic squamous cell carcinoma without evidence of bony metastasis. This hypercalcemia is thought to be mediated through various humoral mechanisms, including parathyroid hormone, parathyroid hormone–like peptides, prostaglandins, vitamin D–like sterols, and osteoclast activating factor 3.

Two radiographic features—enhancement of the tumour on contrast ct, and tumour blush patterns on angiography—have been reported to aid in the differentiation of pancreatic squamous cell carcinoma and classical ductal adenocarcinoma. Both of these radiographic phenomena are probably a result of the hypervascularity of squamous cell carcinoma 3,7,17–19. Fajardo *et al.* 18 reported the use of dynamic ct with a bolus injection of intravenous contrast to examine a patient with pancreatic squamous cell carcinoma. The attenuation of this tumour increased from 35 HU to 61 HU. Sprayregen *et al.* also reported the angiographic features of two cases of squamous cell carcinoma of the pancreas 20. Tumour blushes were present in both cases, and one case showed hypervascularity. He concluded that these features may help to differentiate squamous cell carcinoma from the typical pancreatic adenocarcinoma 20.

Although the foregoing radiologic features are unusual in typical ductal adenocarcinoma of the pancreas and are more suggestive of pancreatic squamous cell carcinoma, hypervascular radiographic features can be seen in other conditions such as cystadenoma and cystadenocarcinoma, adenosquamous carcinoma, hemangioma, angiosarcoma, leiomyosarcoma, and islet cell tumours of the pancreas 3,18. However, squamous cell carcinoma should be included in the differential diagnosis when hypervascularity or a tumour blush is demonstrated in the pancreas 20.

Histology findings characteristic of squamous cell carcinoma of the pancreas frequently reported in the literature include keratinization with eosinophilic cytoplasm on hematoxylin and eosin staining, the formation of whorls or “pearls” with intercellular bridges, and irregularly shaped nests and cords of epithelial cells. Desmoplastic response secondary to ductal obstruction may be a prominent feature 3,4. No criteria exist for the cytologic distinction of metastatic from primary pancreatic squamous cell carcinoma 10, and this situation again emphasizes the importance of searching for another primary source.

Several options for treating pancreatic squamous cell carcinoma are described in the literature. They include surgical resection, chemotherapy, and radiotherapy, with curative resection being the best therapeutic option in the appropriate setting. Unfortunately, most cases (more than 80%) are metastatic or locally advanced at the time of diagnosis, which makes curative tumour resection impossible 12,17. A number of chemotherapeutic options have been tried, including the combination of cisplatin and 5-fluorouracil or vinblastine 4,5. One report described better response to chemoradiotherapy regimens based on gemcitabine 8. Ravry, as quoted by Itani *et al.,* reported an objective response with striking symptomatic improvement with bleomycin in 1 of 2 patients with the squamous cell variety of pancreatic cancer 4; however, no standard chemotherapy regimen has been established. Our gastrointestinal tumour group elected to treat our patient with a combination of gemcitabine and carboplatin on a palliative basis, given the modest efficacy of these drugs in treating squamous cell carcinoma of the lung.

The literature on squamous cell carcinoma has reported a variety of median survivals, with one study showing a median survival of 7 months (range: 6–16 months) for patients who underwent curative resection and 3 months (range: 0.25–9 months) for patients who did not undergo curative resection 5. Within the various studies, the median survival was generally poor, with 1-year and 5-year survival rates of 4.8% and 1% respectively 3,11–13,19. Two reports showed a similar mean survival for the two histologically different pancreatic tumour types, but it would appear that prognosis for the squamous cell type may be worse than that of the usual adenocarcinoma of the pancreas 1–3,11–13,19.

## 4. CONCLUSIONS

Primary pancreatic squamous cell carcinoma is such a rare event that the finding of pure squamous cell carcinoma on pancreatic carcinoma biopsy warrants an extensive workup to rule out the possibility of other more common primary sources, including head-and-neck, lung, and esophagus. The disease is highly aggressive, most often locally advanced or metastatic at diagnosis, and poorly responsive to chemotherapy or radiotherapy; it also has generally poor survival rates. Based on the rare incidence of this histologic subtype of pancreatic carcinoma, advancing diagnosis and treatment will remain an enormous challenge.
